# Individual Responses to Heat Stress: Implications for Hyperthermia and Physical Work Capacity

**DOI:** 10.3389/fphys.2020.541483

**Published:** 2020-09-11

**Authors:** Josh Foster, Simon G. Hodder, Alex B. Lloyd, George Havenith

**Affiliations:** Environmental Ergonomics Research Centre, Loughborough University, Loughborough, United Kingdom

**Keywords:** heat, fitness, sex, age, diabetes, hyperthermia, performance, acclimation

## Abstract

**Background:**

Extreme heat events are increasing in frequency, severity, and duration. It is well known that heat stress can have a negative impact on occupational health and productivity, particularly during physical work. However, there are no up-to-date reviews on how vulnerability to heat changes as a function of individual characteristics in relation to the risk of hyperthermia and work capacity loss. The objective of this narrative review is to examine the role of individual characteristics on the human heat stress response, specifically in relation to hyperthermia risk and productivity loss in hot workplaces. Finally, we aim to generate practical guidance for industrial hygienists considering our findings. Factors included in the analysis were body mass, body surface area to mass ratio, body fat, aerobic fitness and training, heat adaptation, aging, sex, and chronic health conditions.

**Findings:**

We found the relevance of any factor to be dynamic, based on the work-type (fixed pace or relative to fitness level), work intensity (low, moderate, or heavy work), climate type (humidity, clothing vapor resistance), and variable of interest (risk of hyperthermia or likelihood of productivity loss). Heat adaptation, high aerobic fitness, and having a large body mass are the most protective factors during heat exposure. Primary detrimental factors include low fitness, low body mass, and lack of heat adaptation. Aging beyond 50 years, being female, and diabetes are less impactful negative factors, since their independent effect is quite small in well matched participants. Skin surface area to mass ratio, body composition, hypertension, and cardiovascular disease are not strong independent predictors of the heat stress response.

**Conclusion:**

Understanding how individual factors impact responses to heat stress is necessary for the prediction of heat wave impacts on occupational health and work capacity. The recommendations provided in this report could be utilized to help curtail hyperthermia risk and productivity losses induced by heat.

## Introduction

Climate change is increasing the frequency, intensity, and duration of extreme heat events. Consequently, the prevalence of occupational heat stress is also increasing, which reduces the ability of workers to live healthy and productive lives (Flouris et al., 2018a). Most affected are those who work with sun exposure, in non-air-conditioned work spaces, those who perform heavy work, or those who require protective clothing. Sustained, daily elevations in body temperature can increase the risk ofkidney injuries, and is also strongly linked to workplace accident rates (Tawatsupa et al., 2012, 2013). Heat stress also decreases physical work productivity (Wyndham, 1969), since workers must reduce their work output to minimize physiological strain and risk of heat stroke (Miller et al., 2011). The health and productivity implications of workplace heat decreases national economic income (Hübler et al., 2008), an effect exacerbated with climate change (Hsiang et al., 2017).

Although the link between heat stress, health, and performance on the macro level is well established, the biophysical and physiological factors that impact the vulnerability of the *individual worker* is still debated. While past reviews have addressed the impact of some individual characteristics on the heat stress response, an updated synthesis that has practical use is urgently required. Havenith’s report (1985) was extensive for the time, but due to lack of available data the discussion on age and body characteristics were limited, and diabetes was not known to be a relevant factor in thermoregulatory control. Cheung et al. (2000) addressed physiological responses to uncompensable heat stress only, which is relevant in many settings (especially with highly protective clothing) but generally less common than compensable environments (each are defined in the “Clarification of Terms” section). Kenny and Jay (2013) summarized the independent effect of age, sex, and diabetes on the heat stress response as part of a larger review, but their conclusions are drawn mostly from groups matched for all other characteristics apart from that under investigation, rather than the population distribution. They also do not comment on the cardiovascular adjustments to heat stress, which is relevant because workers seem to pace themselves based on their heart rate (HR), a proxy for cardiovascular strain (Miller et al., 2011).

In the present review, we indeed report on individual differences for matched individuals, but also for unmatched groups, which is a better representation of the population distribution. This approach allows for conclusions to be made on a wider scale, facilitating the development of practical advice for policymakers and industrial hygienists. We also recognize the contribution of large, individual lab studies which use heterogeneous groups and multiple regression to document the most relevant factors governing the heat stress response (Havenith et al., 1995b, 1998; Havenith and van Middendorp, 1990; Flouris et al., 2018b; Notley et al., 2019b). These works are addressed throughout this paper, based on their contribution to understanding the influence of each individual factor described below. However, no one single study can answer all the relevant questions needed to determine the importance of any given individual characteristic. The relative importance of each factor changes based on the environment (hot dry or hot humid), work intensity (low, moderate, or high metabolic rate, Table 1), and work type (fixed or self-paced). Equally important to consider is the cardiovascular response to heat (particularly HR), since this can govern perceived work intensity and thus, work output (discussed in the “Physiological and Biophysical Aspects of Heat Transfer” section) (Miller et al., 2011). Any impact a single factor has on work capacity therefore has implications for economic production.

**TABLE 1:** Classification of work intensity according to heat production and type of activity.

Class	Average metabolic rate (with range in brackets)		Examples
	W/m^–2^	W	
Resting	65 (55 to 70)	115 (100 to 125)	Resting, sitting at ease.
Low metabolic rate	100 (70 to 130)	180 (125 to 235)	Light manual work (writing, typing, drawing, sewing, book-keeping); hand and arm work (small bench tools, inspection, assembly or sorting or light materials); arm and leg work (driving vehicle in normal conditions, operating foot switch or pedal); standing drilling (small parts); milling machine (small parts); coil winding; small armature winding; machining with low power tools; walking up to 2.5 km/h.
Moderate metabolic rate	165 (130 to 200)	295 (235 to 360)	Sustained hand and arm work (hammering in nails, filing); arm and leg work (off-road operation of lorries, tractors or construction equipment); arm and trunk work (work with pneumatic hammer, tractor assembly, plastering, intermittent handling of moderately heavy material, weeding, hoeing, picking fruits or vegetables, pushing or pulling lightweight carts, wheelbarrows, walking at a speed of 2.5 km/h to 5.5 km/h).
High metabolic rate	230 (200 to 260)	415 (360 to 465)	Intense arm and trunk work; carrying heavy material; shoveling; sledgehammer work; sawing; planning or chiseling hard wood; hand mowing; digging; walking at a speed of 5.5 km/h to 7 km/h. Pushing or pulling heavily loaded hand carts or wheelbarrows; chipping castings; concrete block laying.
Very high metabolic rate	290 (>260)	520 (>465)	Very intense activity at fast to maximum pace; working with an axe; intense shoveling or digging; climbing stairs, ramp or ladder; walking quickly with small steps; running; walking at a speed greater than 7 km/h.

*Source: International Standards Committee [International Organisation for Standardisation (ISO), 2004].*

The aim of this review is to synthesize the relative importance of individual factors, based on how they can predict the human heat stress response. We base our conclusions on how each factor may be protective against hyperthermia (rises in *T*_*c*_) and losses in physical work output during fixed and self-paced work scenarios.

## Methodology

We chose to perform a narrative review due to (i) concerns that the systematic review process will omit many studies based on strict inclusion/exclusion criteria, and (ii) the broad scope of the present review, which is unsuitable if using the systematic process (Misra and Agarwal, 2018). Articles were obtained by searching relevant keywords into Google Scholar and PubMed databases. The reference list of relevant articles was also scanned for their potential inclusion.

## Clarification of Terms

This section will aim to improve the translation of findings from laboratory studies to real-world working scenarios. To achieve this aim, we present a clarification of terms used throughout this review.

### Core Temperature

The term *T*_*c*_ is used to reflect the global internal temperature of the body. The rectal (typically 10−12 cm beyond the anal sphincter) and/or oesophaeal (typically level with the left atrium) temperatures are the most adopted tissues used to estimate *T*_*c*_. Alternative measurements are intestinal temperature, arterial blood, tympanic, and brain temperature, but each have issues of either cost, invasiveness, logistics during exercise, or accuracy, decreasing their use. A further consideration is that there may be a time lag of 10−30 min for *T*_*c*_ to reflect whole body heat content (Kenny and Jay, 2013).

### Direct Calorimetry

Various thermoreceptors sense temperature variations throughout the body to generate an appropriate effector response (Romanovsky, 2018), with the global internal temperature best represented whole body heat content (Kenny and Jay, 2013). Whole-body heat content can be measured with a *direct calorimeter*, a unique tool which generates data on each heat transfer pathway (evaporative, dry, and respiratory), and in combination with indirect calorimetry to measure metabolic rate, whole body heat storage. Using direct calorimetry, differences in whole body heat storage help to identify inter-individual differences in heat exchange pathways (Larose et al., 2013; Stapleton et al., 2013, 2015; Carter et al., 2014; Kenny et al., 2015; Poirier et al., 2015; Flouris et al., 2018b; Notley et al., 2019b). Due to reasons previously described, the device is primarily limited to cycling exercise in hot dry environments, and with high air flow (to minimize sweat drippage) (Cramer and Jay, 2019). Hence, the environment is considered in each study when drawing conclusions about the data from direct calorimetry.

### Fixed Work Rate

Protocols that require participants to work at a *fixed metabolic rate* simulate a constant work rate, not allowing for self-pacing of exercise intensity (Havenith et al., 1998). This type of activity may reflect work on an assembly line where the work pace is fixed for all. The approach is often used in regression studies to determine what individual factors best predict the heat stress response (Havenith et al., 1995b, 1998; Havenith and van Middendorp, 1990; Cramer and Jay, 2015).

### Activity at a Relative Intensity

The term *relative exercise intensity* means the workload is prescribed based on the individual participant’s maximal work capacity (Havenith et al., 1998; Periard et al., 2012). Here, fitter people will work at a greater metabolic rate than unfit people to achieve equivalent percentage maximum oxygen uptake (*V̇*O_2__*max*_). It stands to reason therefore, that results from studies that use a relative intensity can be used to reflect scenarios where physical work is self-paced. This is supported by evidence of self-pacing during actual physical work in the heat (Wyndham et al., 1965; Morrison et al., 1969; Kalkowsky and Kampmann, 2006; Miller et al., 2011; Bröde et al., 2018). In a laboratory setting, the intensity is normally set as a percentage of *V̇*O_2__*max*_, normally prescribed relative to body mass (ml O_2_⋅kg^–1^⋅min^–1^).

### Compensable and Uncompensable Heat Stress

Environments in the present review are often characterized based on whether they are *compensable* or *uncompensable*. A distinction between compensable and uncompensable heat stress is required since it can have implications for the relevance of individual characteristics. These terms describe if metabolic heat production can be matched by heat loss. In compensable heat stress, enough heat can be lost to the environment so that the body is not in a continuous state of heat gain. In hot working scenarios, compensable heat stress is typically associated with work in an environment with low ambient humidity. With uncompensable heat stress, heat production exceeds heat loss potential in that climate, and the body is in a state of continuous heat gain. Thermal compensability can be determined by estimating required evaporative heat loss (*E*_*req*_) and the maximum evaporative capacity of the environment (*E*_*max*_), determined by the humidity, wind speed, and clothing. A work situation is generally considered compensable if *E*_*max*_ > *E*_*req*_, indicating the environment can accommodate *E*_*req*_ for thermal balance.

### Relating Heart Rate to Physical Work Capacity

Physical work capacity defines the ability of an individual to perform maximal physical work. To support SkBF requirements during work, cardiac output (primarily mediated by HR) increases as a function of the heat stress severity (Rowell, 1974). Because the WHO have classified occupational work intensities based on HR (Table 2; Andersen et al., 1978), HR is considered an integral part of the heat stress response. Moreover, there are a number of large scale field observations showing that workers will pace themselves according to the environmental heat, resulting in a relatively stable working HR regardless of the environmental severity (Morrison et al., 1969; Wyndham, 1973; Vogt et al., 1983; Kalkowsky and Kampmann, 2006; Miller et al., 2011). Since self-pacing is primarily driven by HR (Borg, 1982), those with a more stable and lower HR increase during hot work will likely maintain greater physical work capacity (Jay et al., 2019).

**TABLE 2:** Relative work intensity classification based on heart rate in young adult males.

Work intensity	*V̇*O_2_ (L⋅min^–1^)	% of *V̇*O_2_ max	Heart rate (b⋅min^–1^)
Light	<1.0	<25	<100
Moderate	1.0−1.4	26−50	100−124
Heavy	1.5−2.0	51−75	124−150
Very heavy	>2.0	>75	>150

*Source: Andersen et al. (1978).*

### The Relative Contribution of SkBF to Heat Loss

Control of human *T*_*c*_ relies on delivery of warm blood from the core to the skin surface. Heat loss from the skin surface to the environment can then occur through dry and/or evaporative pathways. Throughout this review, reference is made to adjustments in SkBF with specific factors, but its contribution to overall heat loss should be nuanced relative to the environment. In resting, normothermic conditions, blood is delivered to the skin at a rate of ∼250 ml/min, warming the skin. Heat from the skin surface is then *lost* to the environment (dry heat loss) at a rate similar to metabolic heat *production*, producing heat balance (Charkoudian, 2003). The rate of dry heat loss is therefore modified by SkBF in resting conditions in a cool environment, where it is the primary contributor to overall heat loss. The contribution of dry heat loss (and thus SkBF) to overall heat loss is minimal in hotter conditions due to a narrowing of the skin and air temperature gradient. For instance, at 30°C air temperature, attenuated SkBF causes a faster increase in *T*_*c*_ during activity in the heat, despite similar sweat rates (Balmain et al., 2018a). However, at 35°C air temperature, a reduced SkBF in older participants *did not* increase *T*_*c*_, because dry heat loss was similar in the young and older participants (Havenith et al., 1995b).

Thus, if an individual factor is shown to modify SkBF, this in-of-itself is likely to improve heat loss mainly in conditions permitting high rates of *dry* heat transfer. Such conditions are air temperatures < 30°C, minimal clothing insulation, and high wind speed. All these factors increase rates of dry heat loss from the skin to the environment, rendering an elevation in SkBF beneficial to the heat loss response. A secondary effect of SkBF raising skin temperature is its effect on the saturated vapor pressure on the skin, which increases to a small amount with each degree of increase in skin temperature (Parsons, 2010). This effect increases the vapor pressure gradient from the skin to the environment, increasing sweating efficiency i.e., the proportion off sweat that evaporates, rather than drips from the body (Candas et al., 1979).

### The Relative Contribution of SkBF to Work Capacity

Important to note is the role of SkBF in overall cardiovascular strain, which impacts work tolerance time in the heat. In several studies throughout this review, differences in *absolute* SkBF do not result in different body temperatures, an observation supported and explained by Kenney and Havenith (1993). However, while changes in *absolute* SkBF may not result in a different *T*_*c*_ (and risk of hyperthermia *per se*), such differences can have implications for work capacity, depending on the %HR_*max*_ required to achieve that SkBF (Rowell et al., 1970; Rowell, 1974). For individual factors that reduce maximum cardiac output (i.e., low fitness, age), a similar, or even lower *absolute* level of SkBF can still represent a greater *relative* cardiovascular strain (in terms of %HR_*max*_), which is a major limitation to work capacity in the heat (Drinkwater and Horvath, 1979; Cheung and McLellan, 1998). For example, when comparing young vs. older participants, despite *absolute* SkBF being lower in older participants and no corresponding change in *T*_*c*_ (Havenith et al., 1995b), older people required a similar %HR_*max*_ to achieve their SkBF, placing similar *relative* stress on the cardiac system to meet the combined oxygen demand of locomotion (active muscle tissues) and thermoregulation (skin tissues). The net result would be a similar “cardiovascular strain” despite reduced SkBF requirements.

### The Role of Clothing on the Impact of Individual Factors

Clothing is a pre-requisite of most occupations but varies depending on the level of protection required. Clothing impacts dry and evaporative heat transfer pathways (Havenith et al., 1999; Holmér et al., 1999), such that the potential for heat loss decreases as a function of the total insulation and evaporative resistance of a given ensemble (Potter et al., 2015). Many of the factors discussed in this review impact heat stress vulnerability through adaptation or maladaptation of the sweating response, which impacts the rate of sweat evaporative heat loss. However, with heavy protective clothing (i.e., NBC protective clothing), sweat evaporation is severely diminished, resulting in similar *thermoregulatory* responses between people of different phenotypes/individual characteristics (Cheung and McLellan, 1998). Clearly, heavy protective clothing that creates an *uncompensable* environment changes the relevance of individual characteristics, compared with environments where sweat output impacts heat loss. We differentiate between compensable and uncompensable heat stress throughout this review, but the reader is also directed to an earlier review which focuses on individual factors during uncompensable heat stress exclusively (Cheung et al., 2000).

Data is limited regarding the impact of typical clothing (i.e., for non-specialist situations) on the relevance of individual factors. However, in the present review, the impact of added clothing will be like that of increasing ambient humidity since both *decrease* compensability in a similar way. Studies that create a more uncompensable environment by increasing humidity (i.e., Havenith et al., 1995a) are therefore likely to serve as a proxy for increasing clothing insulation. This relationship is not perfect however, and more studies are required to investigate the importance of individual characteristics in typical, non-specialist work ensembles.

### Determining the Hierarchy of Individual Factors

**FIGURE 1:**
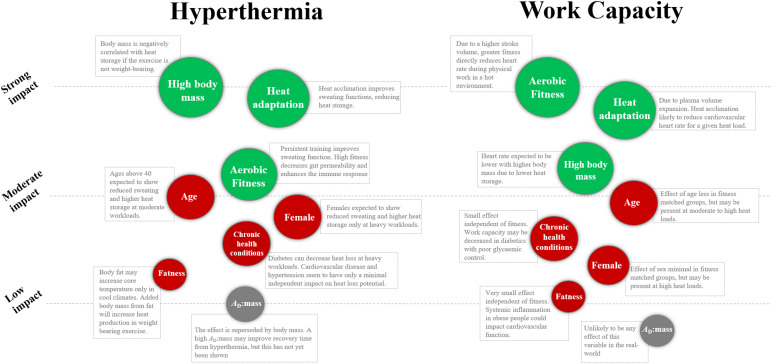
Relative influence of individual characteristics on *T*_*c*_ and work capacity during physical work in the heat. Green variables indicate a positive influence, red variables indicate a negative influence, and the gray variables are deemed not relevant. The orders are based on the authors’ interpretation of the literature, as explained in the clarification of terms section. *A*_*D*_:mass, surface area to mass ratio.

At the end of each section, we use specific terminology to state the overall impact of an individual factor. We also use that terminology to determine the relative importance of an individual factor as shown in Figure 1. If a factor has a “strong” impact on the heat stress response, it is consistently relevant independent of climate type or workload. If a factor has a “moderate” impact on the heat stress response, its relevance is dependent on climate type or workload. If a factor has a “low” impact on the heat stress response, it has minimal *independent* effect on heat stress vulnerability. With that being said, some factors of “low” impact will be secondary to other factors of strong impact, so may still be important screening tools for individual workers.

## Morphological Differences

Morphological factors have been described as key modulators of individual heat stress responses (Havenith et al., 1998; Havenith, 2001a). Factors included for discussion are body mass, the body surface area to mass ratio (*A*_*D*_:mass), and body fat.

### Body Mass

Bergmann’s rule suggests that typically, species originating from colder climates will have a larger body mass than those originating from warm, tropical climates (Bergmann, 1847). The rule tends to apply to modern human beings, but only when extreme differences in climate are apparent (i.e., 50°C of latitude and/or more than 30°C C difference in air temperature) (Foster and Collard, 2013). Here, we show that Bergmann’s rule does not apply to humans, in that heavier people *are not* more vulnerable to heat compared with smaller people. Bergmann’s rule may still apply to extreme geographical changes because (i) absolute fluid requirements are lower in smaller people, and (ii) being heavier increases metabolic heat production if the activity has a considerable weight bearing component i.e., climbing, jogging, etc (Dennis and Noakes, 1999; Marino et al., 2000; Smoljanić et al., 2014). In most occupations, there is minimal weight bearing component and water is not typically in short supply. Below, we discuss the effects of body mass with the assumption that an individual is within a healthy range of body fat.

#### Fixed Work Rate

In humans, specific heat capacity defines the amount of energy required to heat the body by 1°C. The specific heat of most tissue in the body is ∼3.65 kJ⋅kg^–1^, apart from adipose tissue which is ∼2.51 J⋅g^–1^ (Lipkin and Hardy, 1954). Compared with light people, larger people are at an advantage if they work at the same absolute metabolic rate, since their larger heat sink results in more energy being required to raise *T*_*c*_ (Havenith, 1997). Consequently, total body mass below 50 kg has been highlighted as a major risk factor for hot work in general (Wyndham and Heyns, 1973). Body mass has been shown to explain a large portion of the heat stress responses during fixed and relative exercise intensities (Havenith, 1985; Havenith et al., 1995a, b, 1998; Coso et al., 2011; Cramer and Jay, 2014, 2015). Since a larger body mass allows for greater distribution of internal heat (i.e., “heat sink”), Δ*T*_*c*_ is negatively correlated with body mass during hot work (Lind, 1963; Havenith et al., 1995b, 1998; Havenith, 2001a; Gagnon et al., 2009, 2013b; Coso et al., 2011; Cramer and Jay, 2015). During cycling at a fixed work rate, body mass explained ∼40% of Δ*T*_*c*_, where it is negatively correlated i.e., protective (Havenith et al., 1998; Cramer and Jay, 2014). HR was also negatively correlated with body mass during fixed pace work in both dry and humid conditions (Havenith et al., 1995a), indicating a protective impact on physical work capacity. In occupational settings where the workload is externally governed, it can be assumed that heavier people are less vulnerable to heat stress compared with lighter people. However, upon recovery from heat stress, people with a heavy body mass will generally have a slower rate of *T*_*c*_ decrease compared with smaller people (White et al., 1992). This is relevant for occupations that adopt fixed work/rest cycles because heavier people may take longer to recover to their baseline *T*_*c*_.

#### Relative Work Rate

The effect of total body mass was determined in hot-dry and warm-humid environments during exercise at a relative workload (Havenith et al., 1998). During exercise in a compensable environment, body mass explained ∼10% of the Δ*T*_*c*_, where there was a negative association. In the more uncompensable environment, body mass explained 30% of the Δ*T*_*c*_, also with a negative association. However, body mass had no independent effect on HR during relative work in dry or humid heat (Havenith et al., 1995a). Therefore, body mass remains protective against hyperthermia at relative workloads, without a strong impact on HR.

In summary, total body mass has a strong impact on the heat stress response in humans, where it is protective against hyperthermia and increased HR during fixed paced work (Havenith et al., 1998). A high body mass remains protective against hyperthermia during self-paced work, without impacting HR (Havenith et al., 1995a). The findings are unlikely to apply to activities with a heavy weight bearing component.

### Surface Area to Mass Ratio

Allen (1907) rule suggests that homeothermic animals adapted to their thermal environment through evolutionary alterations in the skin surface area to body mass ratio (*A*_*D*_:mass). In short, the rule suggests that a high *A*_*D*_:mass decreases heat gain, due to a larger ratio of cooled tissue (from dry and evaporative heat exchange at the skin) to metabolically active tissue (the body mass reflects this component). Geographical adaptations to heat are evident since humans born in hot climates generally show greater limb length compared with those descending from cold climates (Katzmarzyk and Leonard, 1998; Weinstein, 2005).

The literature examining the human heat stress response *does not* support Allen’s rule. Heavier people mostly have a lower *A*_*D*_:mass than lighter people, because mass and *A*_*D*_ do not increase in direct proportion to one another. Hence, the proportion of *A*_*D*_ in relation to mass typically decreases as mass increases, unless an individual is exceptionally tall and lean. For example, if an individual was 60 kg and 1.8 m (5.9 ft) tall, their *A*_*D*_:mass would be 294 cm^2^⋅kg^–1^. For a heavier person (80 kg) to achieve the same *A*_*D*_:mass, they would need to be 2.2 m (7.2 ft) tall, clearly not a population norm. Ultimately, there is strong collinearity between mass and *A*_*D*_:mass in most population samples, meaning that *A*_*D*_:mass is effectively, another representation of mass itself (White et al., 1992; Havenith, 2001a).

During non-weight bearing activity in the heat, Δ*T*_*c*_ is more related to total body mass compared with *A*_*D*_:mass. Havenith (2001a) reports total mass to be the most relevant characteristics for heating rates during hot work at a fixed metabolic rate, where *A*_*D*_:mass was not a stronger predictor. Moreover, *A*_*D*_:mass generally *increases* as body mass *decreases*, such that a higher *A*_*D*_:mass is associated with a faster Δ*T*_*c*_. It was shown in the last section that a lower body mass decreases heat-sink, which results in an increased Δ*T*_*c*_ for lighter people. Hence, during physical work, participants with a higher *A*_*D*_:mass (smaller people) showed elevated *T*_*c*_’s compared with the heavier people (Havenith et al., 1998; Havenith, 2001a; Cramer and Jay, 2015). Taken together, these findings contradict Allen’s and Bergmann’s rules. An earlier study analyzed sex differences in responses to heat stress, reporting a lower heat gain in females due to their higher *A*_*D*_:mass (Shapiro et al., 1980). However, the interpretation was shown to be erroneous because the heavier males (i.e., lower *A*_*D*_:mass) were working at higher rates of heat production than the lighter females (Havenith, 2001a). In humans, *A*_*D*_:mass only seems relevant for two individuals of the same mass, where increased limb length alters heat exchange with the environment. In environments where air temperature is below skin temperature, having a larger *A*_*D*_ for the same mass *increases* heat loss by convection and radiation. Conversely, when air temperature exceeds skin temperature, more heat will be gained from these dry heat exchange pathways with increasing *A*_*D*_. An increased *A*_*D*_ will also increase *E*_*max*_, which is beneficial in all heat stress conditions, if sweat can evaporate freely i.e., compensable. It is worth noting that occupational heat stress typically involves short, non-steady state heat stress exposures (Vogt et al., 1983), often not allowing time for steady state sweating to occur. In those scenarios where the skin is not wet, dry heat exchange, primarily determined by the gradient between skin and ambient temperature, will become highly relevant. Overall, body mass is the more relevant characteristic during heat stress.

Similar to the last section, total body mass was a stronger predictor of Δ*T*_*c*_ during recovery compared with *A*_*D*_:mass (White et al., 1992). However, that study used cold water immersion during recovery, which is applicable for heat stroke recovery but less commonly adopted in occupational settings. More data on the association between body characteristics during recovery from heat stress in cool and hot air is required as it is more applicable to industry.

Overall, *A*_*D*_:mass *is not* a strong independent predictor of the heat stress response in humans.

### Body Fat

Based on the physical properties of fat tissue (described below), the WHO suggest that being overweight increases vulnerability to hyperthermia during heat stress (Koppe et al., 2004). Body fat can affect the heat stress response in several ways. Firstly, fat tissue has different heat transfer properties compared with muscle (McIntosh and Anderson, 2010). The comparison between these tissues is appropriate in the context of comparing individuals with different body compositions. The properties are shown in Table 3.

**TABLE 3:** Physical properties of fat and skeletal muscle.

	Specific heat capacity, *c* J⋅kg^–1^⋅°C^–1^	Conductivity, *k* W⋅m^–1^⋅°C	Density, *p* kg⋅m^3^	Thermal diffusivity, *a* m^2^⋅s^–1^
**Fat**	2065	0.21	909	1.12E-07
**Skeletal muscle**	3322	0.49	1103	1.34E-07

*Source: McIntosh and Anderson (2010).*

The specific heat capacity (*c*) of a tissue defines the thermal energy required to raise its temperature by 1°C. Fat has a lower value of *c*, which means it requires less thermal energy to raise its temperature. Fat also has a lower value of *k*, which means less heat propagates from the tissue into the blood stream. These aspects intuitively lead to the assumption that body fat independently changes an individual’s vulnerability to heat stress. Importantly however, these values are provided for resting conditions only, not taking into account the fact that during activity, the metabolic heat production of active skeletal muscle will far exceed fat, contributing heavily to whole body heat storage rates (Jay et al., 2007; Kenny and Jay, 2013). However, body fat also increases passive mass carried, another form of load carriage, which elevates metabolic heat production for a given task (Pandolf et al., 1977). Finally, obese humans typically have greater levels of systemic inflammation (Fontana et al., 2007), which, *theoretically*, may predispose this group to heat stroke (Chin et al., 2006). The above factors intuitively lead to the assumption that body fat independently increases vulnerability to heat stress (Havenith, 1997).

A primary supporting article for this assumption showed that overweight army trainees were 70% more likely to develop heat illness in basic training compared with those who have a healthy body fat (Bedno et al., 2010). However, the increased risk of heat illness could have been be due to reduced fitness levels in the overweight recruits (Mondal and Mishra, 2017), which was not accounted for in that study. The extra passive mass carried during the weight bearing activity could have also increased metabolic heat production, contributing to the risk of heat exhaustion. In lab studies which do control for these variables, researchers generally cannot isolate an independent effect of body fat on the heat stress response (Haymes et al., 1974; Havenith et al., 1998; Jay et al., 2011; Adams et al., 2015). Work using a multiple regression approach could not identify body fat as a significant predictor for the heat stress response in dry or humid heat conditions, and at either a fixed or relative exercise intensity (Havenith et al., 1998). Similar findings have been documented in studies using independent matched groups designsin hot (Adams et al., 2015) or warm conditions (Jay et al., 2011). Mechanistically, the potentially insulating effect of fat seems to be outweighed by unimpeded blood flow to the skin surface across the fat layer i.e., blood flow provides a convective short-cut for heat-transport through the fat tissue (Havenith, 2001b). We acknowledge evidence of reduced SkBF in obese vs. lean individuals exercising in the heat (Vroman et al., 1983), but given that sweating is not impaired in obese individuals, any influence of fat on SkBF is unlikely to pose significant increases in risk of heat illness (Dervis et al., 2016). On the population level (i.e., not fitness matched), obese individuals are expected to show an increased HR of 20−30 b⋅min^–1^ during work in hot conditions compared with those of normal body fat (Bar-Or et al., 1969; Haymes et al., 1975).

During exercise in cool conditions, the insulative effect of body fat can increase heat storage rates. At a relative intensity, body fat independently explained 26% of the *T*_*c*_ response in cold conditions, but not in hot conditions (Havenith et al., 1998). Another study found that large differences in body fat of ∼21% increased heat gain during fixed intensity exercise in warm conditions (Dervis et al., 2016). Therefore, it appears that the difference in heat storage between high and low-fat populations increases as the temperature decreases, because the insulative effect of fat takes precedence in colder conditions. Although less specific, the body mass index (BMI) may be a practical guideline when formulating employment standards for hot work. Work using ROC curve analysis suggest an upper threshold for BMI of 26 kg⋅m^2^ for protection against heat illness (Flouris et al., 2018b). Since being underweight is also problematic for heat storage capacity, we advise a lower limit of 18.5 kg⋅m^2^, following WHO guidelines.

Overall, body fat *is not* a strong independent predictor of the heat stress response, but in cool conditions, will likely cause faster elevations in *T*_*c*_ at heavy workloads. On a population level, obese individuals are likely to experience a higher HR and produce less physical work during heat stress.

### Summary

In the normal range of body fat, total body mass can be a strong predictor of the heat stress response, where it is protective during hot work. The beneficial effect of a high mass is greater during uncompensable heat stress compared with compensable heat stress. The *A*_*D*_:mass is not a strong predictor of the heat stress response, and is superseded by body mass. On a population level, individuals with high adiposity do not typically show a different Δ*T*_*c*_ than leaner males in hot conditions but may have increased HR’s due to (on average) lower fitness levels and more passive mass carried. The independent effect of body fat on thermophysiological responses to exercise are displayed in Table 4.

**TABLE 4:** Overview of data relating to the effect of body fat on the heat stress response.

Source	*n*	Fitness matched?	Condition	Work-type	Absolute difference in body fat%	Sweat threshold (°C)	Sweat rate	Baseline Core temperature (°C)	Peak Core temperature (°C)	Cardiovascular strain
Bar-Or et al., 1969	9	No	37°C/15%	Walking on a level treadmill 4.8 km/h	15%	−	↑ 60%	NS	↑ 0.40°C	↑ 20 b/min
Haymes et al., 1975	12 (children)	No	48°C/20%	Walking on a level treadmill 4.8 km/h	15%	−	NS	NS	↑ 0.40°C	↑ 30 b/min
Vroman et al., 1983	10	Yes	38°C/17%	Cycling at 50% *V̇*O_2max_ (ml/fat free mass in kg^–1^/min^–1^).	16%	−	−	NS	NS	
Dervis et al., 2016	16	Yes	28°C/26%	Cycling at a heat production of 550 W	21%	−	NS	NS	↑ 0.20°C	−
				Cycling at a heat production of 7.5 W/kg lean body mass		−	↓ 34%	NS	↓ 28%	−
Adams et al., 2015	20	No	40°C/30%	Cycling a heat production of 300 W	16%	−	NS	NS	NS	NS
				Cycling at a heat production of 175 W/m^–2^		−	NS	NS	NS	NS
Limbaugh et al., 2013	17	Yes	30°C/40%	Cycling at 66 W external work	13%	NS	NS	NS	NS	NS

*HR, heart rate; b/min, heart beats per minute; n, number of subjects; V̇O_2max_, maximal oxygen consumption; W/m^–2^, Watts per meters squared; NS, not statistically significant; ↑, increased; ↓ decreased; −, not reported.*

### Practical Advice

1. If employment standards for hot work are utilized based on body type, they should be based on body mass, and not *A*_*D*_:mass. Heavier people with a normal body fat are at less risk of hyperthermia if the workload is fixed.
2. Previous research suggests those under 50 kg should not perform physical work in the heat.
3. For general purposes, the BMI should be within 18.5 and 26 kg⋅m^2^.

## Aerobic Fitness and Training

Exercise training evokes a plethora of adaptations relevant to thermoregulation, such as increased cardiac function, plasma volume, and microvascular function (Hellsten and Nyberg, 2016). It is logical to assume, therefore, that the physiological adaptations to endurance training directly improve thermoregulatory and cardiovascular performance during heat stress. In exercise physiology, *V̇*O_2__*max*_ is the most used index of aerobic fitness. It is most commonly measured through analysis of expired air during maximal aerobic exercise, but can be predicted during cycling or treadmill exercise based on the power output, and speed and grade, respectively (American College of Sports Medicine [ACSM], 2013; Ludlow and Weyand, 2017). In line with exercise physiology literature, we use *V̇*O_2__*max*_ to categorize aerobic fitness levels.

### Cardiovascular Adaptations

Endurance training increases cardiovascular and thermoregulatory stability during exercise (Ekblom et al., 1968; Ho et al., 1997; Convertino, 1991; Cramer et al., 2012). In older (previously sedentary) participants, physical training decreased *T*_*c*_ and HR during fixed work in the heat, without alterations in body characteristics (Ho et al., 1997). The data indicate that training increased SkBF and plasma volume for the same fixed workload. Endurance training typically increases SkBF for a given *T*_*c*_ and can activate vasodilation for a lower *T*_*c*_ (Roberts et al., 1977; Thomas et al., 1999; Beaudin et al., 2009; Simmons et al., 2011), results not seen with resistance training (Thomas et al., 1999). The increase in SkBF is explained by an expansion of blood volume and increased cardiac output (Simmons et al., 2011), and increases basal production of nitric oxide, an endothelium derived vasodilatory compound (Kingwell et al., 1997; Holowatz and Kenney, 2010).

### Sweating Adaptations

In addition to the cardiovascular adaptations that are beneficial during hot work, exercise training also enhances sweating function. For instance, endurance training can reduce the *T*_*c*_ threshold for the onset of sweating (Nadel et al., 1974; Henane et al., 1977; Roberts et al., 1977) similar to the effects seen from heat adaptation, but to a lesser extent. Modeling the response based on available literature, Havenith (2001b) suggests that a training-induced increase in *V̇*O_2__*max*_ by 12−17% will reduce the sweat onset threshold by 0.1°C, although reductions up to 0.4°C have been reported in a low sample size (Henane et al., 1977). With exercise training, there are increases in sweat output for the same *T*_*c*_ increase, in addition to elevations in maximal sweat output (Nadel et al., 1974; Henane et al., 1977; Roberts et al., 1977). Most recently, 8 weeks exercise training (*V̇*O_2__*max*_ increase from 46 to 52 ml/kg/min) increased local sweat rate, and thus skin wettedness from 72 to 85% surface area (Ravanelli et al., 2018). The increased sweating function in the above studies may not be related to aerobic fitness *per se*, but more due to frequent and persistent rises in *T*_*c*_ due to the training itself, evoking a mild heat adaptation (Ravanelli et al., 2020).

### Climate Type

At a relative intensity, fitter people had a slower increase in *T*_*c*_ in both cool and hot-dry climates, but a faster increase in *T*_*c*_ in very humid heat (Havenith et al., 1998). Thus, when evaporative heat loss is limited by high humidity, the greater heat produced by fitter people makes them more vulnerable to heat. While a similar study documents equivalent thermoregulatory patterns between trained and untrained males in humid heat (Periard et al., 2012), the water vapor pressure was ∼1 kPa higher in the study of Havenith et al. (1998), suggesting an upper critical kPa where a higher metabolic rate can be compensated for by increasing sweat rates. When fully uncompensable conditions are simulated with NBC clothing, fitness has no impact on thermometric responses (Cheung and McLellan, 1998). The above data suggests that impermeable clothing, or an ambient kPa of ∼4 kPa will likely negate any beneficial effect of aerobic fitness on thermoregulatory function. The true upper threshold will depend on the skin temperature and the evaporative resistance of any clothing ensemble.

### Studies Using Multiple Regression

Some studies used multiple regression analysis to determine what variables explain the thermoregulatory responses to heat (Havenith et al., 1995a, 1998; Cramer and Jay, 2015; Flouris et al., 2018b; Notley et al., 2019b). In a temperate environment, the exercising metabolic rate (in W/kg) explained ∼50% of the heat storage during fixed intensity exercise, with aerobic fitness explaining only a further 1% (Cramer and Jay, 2015). It is worth noting that this work used forward entry stepwise regression without interpretation of standardized regression coefficients, which are useful when comparing the relative contribution of individuals parameters that have different units. Therefore, the impact of *V̇*O_2__*max*_ on its own could have been higher 1%. During fixed intensity exercise with dry heat stress, aerobic fitness explained 17−25% of Δ*T*_*c*_, where there was a negative association (Notley et al., 2019b). In humid heat and at a fixed intensity, *T*_*c*_ was negatively associated with fitness, suggesting that fitness has a protective impact (Havenith et al., 1998). In that study, fitness was poorly associated with heat storage in dry heat, but this is likely because *T*_*c*_ was only mildly elevated in that condition. During fixed intensity work, HR was negatively correlated with absolute VO_2__*max*_ in dry and humid heat stress, indicating that those with a higher fitness level will be less vulnerable to losses in physical work capacity independant of the climate type (Havenith et al., 1995a).

Using ROC curve analysis, it was shown that *V̇*O_2__*max*_ thresholds of ≤36.5 and 30 ml⋅kg^–1^⋅min^–1^ be used to identify vulnerable males and females, respectively (Flouris et al., 2018b). Although the fitness requirement for females may be lower, this is explained by the fact they were exercising at a lower absolute heat production. In summary, the impact of fitness on the *T*_*c*_ response is determined by work type (fixed or relative workload) and whether the environment is compensable or uncompensable (see Clarification of terms for definition).

### Tolerance to Heat

An important consideration with respect to fitness level is the effect this has on heat tolerance since this has implications primarily for work capacity. We define heat tolerance as the maximum exposure duration to hot working conditions, which is dictated by global cardiovascular stress and the *T*_*c*_ rise (Cheung and McLellan, 1998). Compared with unfit adults, fitter individuals (*V̇*O_2__*max*_ 62 vs. 40 ml/kg/min) show a lower HR (∼30 b/min) and perceived exertion while cycling at a fixed heat production (Cramer et al., 2012). In the event workers can self-pace, fitter people have an increased physical work capacity for the same HR (Periard et al., 2012). In support, one study found that the Δ*T*_*c*_ was not different between fit and unfit males during uncompensable heat stress, but the fitter individuals had a longer tolerance time, probably explained by their lower HR (Cheung and McLellan, 1998). Another factor relevant to occupational heat stress is that fitter people may have a lower psychological stress for the same rate of heat storage (Tikuisis et al., 2002). A higher fitness level also infers a greater resistance to lipopolysaccharide leakage from the gut (Selkirk et al., 2008), greater cellular tolerance to hyperthermia (Yamada et al., 2008), and a curtailed release of stress hormones (norepinephrine, ACTH) at a given Δ*T*_*c*_ (Wright et al., 2010).

### Summary

Aerobic fitness and endurance training can have a strong impact on heat tolerance and is an important factor for determining work capacity and health during hot work. While research points toward improved sweating function with high fitness, the major benefits stem from an increased cardiovascular function. During fixed intensity work, fitness is associated with a lower rate of heat storage and improved cardiovascular stability. During relative intensity (self-paced) work, fitter people can work harder for an equivalent HR and perceived effort compared with unfit people. Working at a relative intensity (i.e., based on fitness) may elevate the risk of hyperthermia in fitter people if evaporation is impeded by high humidity or protective clothing. The heat stress responses in fit vs. unfit people are shown in Table 5.

**TABLE 5:** Overview of data relating to the effect of aerobic fitness and training on the heat stress response.

Source	Design	n	Condition	Work-type	*V̇*O_2max_ (ml/kg/min)	Difference in *V̇*O_2max_	Sweat threshold (°C)	Baseline Core temperature (°C)	Peak Core temperature (°C)	Cardiovascular strain
Nadel et al., 1974	Training studies (*within subjects).* Sweat threshold test pre- and post-training	6	23.5°C	15 min cycling at 60% *V̇*O_2max_	45 vs. 38	17%	↓ 0.20	−	−	−
Henane et al., 1977		3	30°C/40% to 45°C/24% (i.e., 6°C/min^–1^)	Passive heating for sweating tests	48 vs. 41	↑ 18%	↓ 0.1−0.4	↓ 0.40	NS	−
Drinkwater et al., 1976	Independent groups	12	28°C/45%	100 min walking at 30% *V̇*O_2max_	49 vs. 40	↑ 23%	−	NS	NS	NS
			35°C/65%							
			48°C/10%	50 min walking at 30% *V̇*O_2max_						
Houmard et al., 1990	Training study (*within subjects).* Heat tolerance test pre- and post-training	9	40°C/27%	90 min walking or jogging at 50% *V̇*O_2max_	58. No increase from training.	0%	−	−	NS	↓ with training. Final heart rate 10 b/min less
Cheung and McLellan, 1998	Independent groups. Subjects donned NBC clothing	15	40°C/30%.	60 min walk at 3.5 km/h^–1^	60 vs. 46	↑ 30%	−	NS	NS	↓ in the fitter group. Final heart rate 10 b/min less despite greater tolerance time
Jay et al., 2011	Independent groups	14	24°C/24%	Cycling at 60% *V̇*O_2peak_	40 vs. 60	↑ 49%	↓ 0.40	↓ 0.30	↑ 0.54	−
				Cycling at ∼276 W/m^2^			↓ 0.30	↓ 0.20	NS	−
Periard et al., 2012	Independent groups	16	40°C/50%	Cycling at 60% *V̇*O_2max_	4 vs. 5 l/min	↑ 25%	−	−	NS.	NS
Cramer et al., 2012	Independent groups	21	24°C/30%	Cycling at 60% *V̇*O_2max_ for 60 min	40 vs. 62	↑ 55%	−	↓ 0.30	↑ 0.55	NS
Ravanelli et al., 2018	Training study	8	38°C/65%	Passive exposure. Humidity ramp protocol	46 vs. 52	↑ 14%	−	↓ 0.30	↓ 0.20	↓ with training. Average HR ↓ by 10 b/min

*HR, heart rate; b/min, heart beats per minute; n, number of subjects; V̇O_2max_, maximal oxygen consumption; W/m^–2^, Watts per meters squared; NS, not statistically significant; T_c_, core body temperature; ↑, increased; ↓ decreased; −, not reported.*

### Practical Advice

• For fixed paced physical work in the heat, fitter people will typically be at reduced risk of hyperthermia and productivity loss.
• The benefits of fitness on *T*_*c*_ will be minimized, and even reversed, during uncompensable heat stress i.e., with heavy protective clothing, or in very humid environments (∼ 4 kPa). Even in those conditions, cardiovascular stability, and overall tolerance is likely to be improved with high fitness.
• For self-paced physical work in the heat, fitter people will typically have higher work output, but this could lead to a higher *T*_*c*_ if workers are unacclimatized or inexperienced. Monitoring of all workers is recommended regardless of fitness level.
• During heavy work, those with a *V̇*O_2__*max*_ (ml/kg/min) < 36.5 are more at risk of a higher *T*_*c*_ than those above this threshold.
• During moderate work, those with a *V̇*O_2__*max*_ (ml/kg/min) < 30 are more at risk of a higher *T*_*c*_ than those above this threshold.

## Heat Adaptation

When body tissues are repeatedly exposed to a higher temperature than normal, they adapt to that stress so they can better cope with the physiological demand during future exposures. Adaptation to heat is a reversible phenomenon which begins at a genetic level, manifests to a cellular level, and eventually results in whole body physiological adaptations. Knowledge pertaining to the cardiovascular (Taylor, 2014; Périard et al., 2016), epigenetic (Horowitz, 2014, 2016), and performance (Périard et al., 2015; Tyler et al., 2016) adaptations to heat acclimation have been reported in considerable depth. The reader is also directed to an article describing the early use and development of natural (acclimatization) and artificial (acclimation) adaptation in the mining industry (Schneider, 2016), since it has specific occupational relevance. In this section, we provide a summary of the most relevant information which can inform guidance.

### A Historical View of Heat Adaptation

Scientific appreciation of man’s adaptability to heat can be traced back to 1768. Observing the adaptability of European’s to hot climates, James Lind remarks on behavioral adaptations such as reduced appetite, and changing exposure time by seeking *“repose”* during the heat of the day (Lind, 1768). However, it was not until the early 20^*th*^ century where the study of man’s physiological response to heat adaptation emerged. The following studies form most of the fundamental knowledge in this area (Shaklee, 1917; Dill et al., 1938; Henschel et al., 1943; Robinson et al., 1943, 1965; Eichna et al., 1945; Ladell, 1951; Wyndham, 1951; Hellon et al., 1956a; Wyndham and Jacobs, 1957; Piwonka et al., 1965; Piwonka and Robinson, 1967). Shaklee (1917) published a report on the adaptability of monkeys to heat exposure, and postulated that “*If the monkey can become adapted to life in the tropical sun, man could more readily become adapted*.” He found that the rectal temperature of monkeys exposed to heat was ≥40°C (and sometimes fatal) for the first 2 weeks but was always <40°C for the next 5 months of heat exposure, indicating that most adaptation occurs in the first 2 weeks. He went on to study his own adaptation to heat over a period of 6 months and concluded anecdotally that “*Healthy white men may be more readily acclimatized to the conditions named, that is, to the tropical climate at its worst.”* On that note, potential adaptability to heat does not depend on ethnic origin (Taylor, 2014). In the 1930’s, evidence of decreased sweat-induced ion loss throughout the course of heat exposures was one of the first seminal findings (Dill et al., 1933, 1938). Increased ion reabsorption from sweat glands results in more dilute sweat (Chinevere et al., 2008), which reduces the risk of health issues linked to electrolyte depletion. In the 1940’s, evidence of decreased physiological throughout the course of heat adaptation began to emerge, which is linked to increased sweat rates, and decreased HR and *T*_*c*_ (Henschel et al., 1943; Robinson et al., 1943; Horvath and Shelley, 1946). That work used fixed work rates throughout the daily exposures, and generally found that the work was less taxing on the thermoregulatory and cardiovascular system as acquired heat tolerance developed. Henschel et al. (1943) also found the decay of acquired heat adaptation was ∼3 weeks, a notion supported by modern-day literature. In the 1950’s, more precise data on the adaptation of the sweat rate/*T*_*c*_ relation, as well as the cardiovascular adaptations, such as skin and central blood flow, and cardiac output, which ultimately leads to a reduced HR, began to emerge (Ladell, 1951; Wyndham, 1951; Wyndham et al., 1954b; Wyndham and Jacobs, 1957). After the 1960’s, the individual variation in human adaptability to heat was explored. Generally, the scope for heat adaptation does not seem to depend on chronological age (Robinson et al., 1965; Wagner et al., 1972), sex (Hertig et al., 1963; Frye et al., 1982), or physical fitness (Piwonka and Robinson, 1967; Cheung and McLellan, 1998). This concept is highly relevant to occupational heat exposure because most types of people can physiologically adapt to work in the heat.

### Physiological Adaptations

The extent to which an individual has become heat adapted can be determined through changes in specific physiological, behavioral, and biochemical characteristics. Périard et al. (2015) summarized twenty-five physiological adaptations which occur throughout heat acclimation and the time course for their attainment. Familiar indices include a lower exercising HR, *T*_*c*_ and *T*_*sk*_ at rest and during exercise, an earlier sweating onset and an increased sweat rate for a given *T*_*c*_ (Havenith, 2001b). A plasma volume expansion is a major adaptation which typically peaks after the first week of acclimation (Périard et al., 2016). This adaptation improves cardiovascular stability by increasing vascular filling pressure (Senay et al., 1976) and the specific heat content of blood (Blake et al., 2000). These physiological adaptations allow for improved work performance and comfort during heat stress (Cheung and McLellan, 1998; Lorenzo et al., 2010; Burk et al., 2012; Willmott et al., 2016; James et al., 2017). In support, an early study showed that the risk of syncope during physical work in the heat was due to excessive global cardiovascular strain, but the risk declined throughout the course of acclimation (Eichna et al., 1945). Modeling the sweating adaptation, Havenith (2001b) calculated that acclimation has beneficial effects in terms of (i) reducing sweating onset threshold, and (ii) it can increase the maximum sweat output for the same *T*_*c*_ (see Table 6). Recent work also shows a redistribution of sweat rate toward the limbs, compared to the torso and back with heat acclimation (Smith and Havenith, 2019). Importantly, in an environment which impedes sweat evaporation (i.e., with NBC clothing), heat acclimatized people will typically lose more sweat for the same rate of heat storage, accelerating dehydration (Wyndham et al., 1954a; Cheung and McLellan, 1998).

**TABLE 6:** Overview of data relating to the effect of acclimation on the heat stress response. Participants are young adult males, unless stated otherwise.

Source	Participants (n)	Acclimation duration and environment.	Daily protocol/test of acclimation status	Sweat rate	Baseline Core temperature (°C)	Peak Core temperature (°C)	Sweat threshold (°C)	Cardiovascular strain
Robinson et al., 1943	Young adult (5)	10−23 days. 40°C/23%	Treadmill walking at 5.6 km/h at ∼ 5% incline for 60 to 240 min	↑ 25%	−	↓ 1°C	−	↓∼28 b/min
Wyndham, 1951	13 vs. 353 non-acclimated.	2−3 weeks. ∼47°C, 20%	12 steps/min for 2.5 h (30 min work, 30 min rest)	↑ 54%	−	↓∼0.20−0.5°C	−	↓∼26 b/min
Ladell, 1951	Young adult (17)	9 days. 38°C/77%	12−24 steps/min for 2.5 h (5 min work, 15 min rest)	↑ 48%	↓ 0.29°C	↓ 0.36°C	↓ 0.13°C	↓∼10 b/min
Hertig et al., 1963	Young adult females (5)	10 days at 45°C/20%	Treadmill walking at 4.8 km/h for 2 h	↑ 27%	−	↓ 0.9°C	−	↓∼35 b/min
	Young adult females (4)	10 days at 45°C/50%	Treadmill walking at 5.6 km/h for 2 h	↑ 15%	−	↓ 0.6°C	−	↓∼15 b/min
Robinson et al., 1965	Older (4)	5−13 days. 50°C/23%	Treadmill walking at 5.6 km/h at ∼ 5% incline for 65 to 85 min	↑ 12%	−	↓ 0.9°C	−	↓∼35 b/min
Garden et al., 1966	Young adult (12)	9 days. 36°C/80%	Treadmill walking at 5.6 km/h for 50 min	↑ 17%	NS	NS	−	NS
	Young adult (13)	9 days. 36°C/80%	Treadmill walking at 5.6 km/h for 80 min	↑ 21%	↓ 0.31°C	↓ 0.35°C	−	↓∼15 b/min
	Young adult (13)	9 days. 36°C/80%	Treadmill walking at 5.6 km/h for 100 min	↑ 25%	↓ 0.32°C	↓ 0.2°C	−	↓∼10 b/min
Piwonka and Robinson, 1967	Young adults of high fitness (5)	5 days 50°C/15−20%	5.6 km/h at ∼ 5.6% incline for 85 min	↑ 11%	−	↓ 0.2°C	−	↓∼20 b/min
Gonzalez et al., 1974	Young adult (6)	6 days. 40°C/39%	Cycling at 25% *V̇*O_2max_ for 40 mins	↑ 40%	↓∼0.15°C	↓∼0.4°C	↓ 0.5°C	↓∼5−10 b/min
Henane and Bittel, 1975	Young adult (12)	9 days. 45°C, 24%	Passive exposure at 45°C, 24%	↑ 24%	↓ 0.30°C	↓∼0.40°C	↓ 0.27°C	−
Havenith and van Middendorp, 1986	Young adult, mixed fitness (4)	7 days. 40°C, 20%	Work/rest cycling to regulate *T*_*c*_ at 38.3°C for 2 h	↑ 18%	−	↓ 0.25°C	↓ 0.5°C	↓∼10−14 b/min
Cheung and McLellan, 1998	Young adult, moderate fitness (7)	5 days per week for 2 weeks, i.e., 10 sessions at 40°C/20%	Walking at 40°C/20%. Fully uncompensable due to clothing	↑ 28%	NS	NS	−	↓∼8 b/min
	Young adult, high fitness (8)			↑ 17%	NS	NS	−	↓∼10 b/min
Inoue et al., 1999	Young adult, moderate fitness (5)	8 days at 43°C/30%	Cycling at 35% *V̇*O_2max_ for 90 mins	NS	−	↓ 0.30°C	−	%HRmax ↓ by ∼10% in all groups
	Older, high fitness (4)					↓ 0.40°C	−	
	Older, low fitness (5)					↓ 0.50°C	−	
Garrett et al., 2009	Young adult, moderate fitness (10)	5 days at 39.5°C/60%	Cycling at 40% peak power output in 35°C/60%	−	NS	↓ 0.30°C	−	↓∼13 b/min
Daanen et al., 2011	Young adult, high fitness (15)	9 days at 35°C/29%. Then 3 days at 41°C/33%	Cycling at 45% *V̇*O_2max_ for 60 mins	NS	↓ 0.12°C	↓ 0.17°C	−	↓∼10 b/min
Gibson et al., 2015	Young adult, moderate fitness (8)	5 days at 40°C/39%	Cycling at 50% *V̇*O_2peak_ for 90 mins	↑ 23%	↓ 0.29°C	NS	−	↓∼9 b/min
	Young adult, moderate fitness (8)	10 days at 40°C/39%		↑ 32%	↓ 0.33°C	NS	−	↓∼8 b/min
	Young adult, moderate fitness (8)	5 days at 40°C/39%	Cycling at 65% *V̇*O_2peak_ until *T*_*c*_ reached 38.5°C. Work/rest then adjusted to keep *T*_*c*_ constant	↑ 17%	NS	NS	−	↓∼12 b/min
	Young adult, moderate fitness (8)	10 days at 40°C/39%		↑ 26%	↓ 0.09°C	NS	−	↓∼11 b/min

*b/min, heart beats per minute; n, number of subjects; V̇O_2max_, maximal oxygen consumption; NS, not statistically significant; T_c_, core body temperature; ↑, increased; ↓ decreased; −, not reported.*

### Time Course of Adaptation and Decay

Heat acclimation programs are normally prescribed as short (<7 days), medium (8−12 days) or long (>14 days) durations (Garrett et al., 2011). Generally, the reductions in baseline and exercising *T*_*c*_, *T*_*sk*_, and HR occur after only 4−6 days, while a full adaptation of the sweating response requires ∼12−14 days (Périard et al., 2015). Consequently, the ergogenic effects are maximized in line with improvements in the sweating function, owing to greater skin wetness, and an elevated *E*_*max*_ (Fox et al., 1964). The time-course for the decay of heat acclimation has been addressed in several reviews (Armstrong and Maresh, 1991; Pandolf, 1998; Garrett et al., 2011; Daanen et al., 2018). After a heat acclimation, studies suggest that the adaptations to HR and *T*_*c*_ are lost at a rate of ∼2.5% per day of absence from the heat (Daanen et al., 2018). The general conclusion is that humans return to a pre-acclimation phenotype within 3-weeks of absence from the heat, characterized by a return of sweating responses back to baseline levels (Armstrong and Maresh, 1991; Poirier et al., 2015). Following short-term (5-day) heat acclimation, adaptations to exercising HR and *T*_*c*_ were maintained after 1 week but lost after 2 weeks (Garrett et al., 2009). Isothermic protocols ensure the participant’s *T*_*c*_ is consistent throughout the program, which is preferable to a constant daily work rate. Full adaptation may take place a while after the heat acclimation program itself, as shown by lower resting *T*_*c*_ by ∼0.5°C, 6 days after a nine-day program (Daanen et al., 2011). As observed in the German coal mines, new workers should be paired with a more experienced worker during the initial days of exposure to learn optimal pacing and drinking behaviors (Kalkowsky and Kampmann, 2006). Early work demonstrates a memory feature with heat adaptation, since pre-acclimatized workers took only two days to return to an acclimatized phenotype after a 6-day period of working in cool conditions (Wyndham and Jacobs, 1957). Furthermore, Weller et al. (2007) showed that only 2 and 4 days of heat acclimation was required for re-acclimation following 12 and 26 days of non-exposure to heat stress, respectively. A recent systematic review and meta-analysis showed that heat-reacclimation occurs 8−12 times faster than the process of heat acclimation decay (Daanen et al., 2018). In practical terms, this means that heat acclimation can be maintained relatively simply in workers who have previously undergone a recent procedure of heat acclimation.

### Summary

Adaptation to heat can have a strong impact on the heat stress response, inferring a physiological advantage when sweat evaporation is possible. Strong evidence supports that short-term heat acclimation (<7 days) is beneficial for those required to work in heat stress conditions, reflected by a lower *T*_*c*_ and HR compared with pre-acclimation. Long-term heat acclimation (>14 days) provides further benefit due to adaptation of the sweating mechanism and acquired cellular tolerance to hyperthermia. Evidence of heat acclimation memory suggests that a rapid re-acclimation is likely in individuals previously exposed to long-term heat acclimation. The heat stress responses in non-acclimated and acclimated individuals are shown in Table 6.

### Practical Advice

Advice for natural adaptation:

• Natural acclimatization to hot work will typically occur over 14−30 days.
• Unacclimatized workers should be considered more at risk and be monitored frequently during acclimatization.
• New workers will typically benefit from working with someone more experienced during acclimatization.
• New workers should try to adopt similar fluid replacement behaviors and pacing strategies to more experienced workers.

Advice for laboratory/artificial adaptation:

• Acclimation should be 5-days minimum, with full adaptation taking place over 14-days.
• Adaptations to heat will be lost after ∼3 weeks no heat exposure, but re-acclimatization will typically only take 3−4 days i.e., workers do not have to go through the full 14-day process twice.

General considerations:

• The positive effect of acclimation/acclimatization will be lower if sweat evaporation is impeded with impermeable protective clothing, or in very humid environments.
• Acclimation/acclimatization increases sweat output, so may result in greater body fluid losses, especially in severe environments, such as those noted above.

## Aging

Aging is accompanied by several physiological changes which are relevant to human heat stress vulnerability. In general, the changes are maladaptive for thermoregulation, and include a hampered cardiovascular function (Minson et al., 1998; Betik and Hepple, 2008), sweat gland output (Hellon and Lind, 1956; Anderson and Kenney, 1987; Kenney and Fowler, 1988) and reduced thermal perceptual sensitivity (Dufour and Candas, 2007; Inoue et al., 2016; Coull et al., 2017). While there is overwhelming evidence that pre-frail and frail elderly people account for most of the mortality/morbidity statistics during heat waves (Kenney et al., 2014), the relative impact of age on heat stress responses to physical work needs a general clarification, especially as the effective retirement age in most countries has been rising since 2000. Compared with young adults, aging has been shown to reduce heat loss capacity as early as age 40, primarily through a reduction in whole-body sweat losses for certain work-loads (Larose et al., 2013). These findings help explain the documented link between age and heat stroke risk in Bantu miners (Strydom, 1971). Analysis from that work showed those over the age of 40 accounted for over 50% of fatal heat stroke and 25% of non-fatal heat stroke cases, despite accounting for less than 10% of the total working population (Strydom, 1971).

### Cardiovascular Maladaptation With Age

Seminal work in the 1960’s showed that sustained increases in skin temperature can result in a doubling of cardiac output and resting HR (Rowell et al., 1969). Responses like this, which are accompanied by large reductions in peripheral resistance, cause a re-distribution of blood flow from the core to the cutaneous vascular beds for heat dissipation (Rowell et al., 1969). Since that work, numerous studies compared responses of young and older adults to heat stress. During passive, uncompensable heat stress, elderly people (aged ≥ 65 years) showed a 33% reduction in stroke volume and cardiac output, accounting for 53% reduction in total SkBF compared with young adults (Minson et al., 1998). That healthy aging reduces stroke volume is not a consistent finding however (Gagnon et al., 2016), and the explanation for the disparity between studies remains unclear. With advancing age, many studies have shown that vasoreactivity is reduced during heat stress (Holowatz et al., 2003; Stanhewicz et al., 2015, 2017). Nitric oxide is an important vasodilatory molecule but its concentration within the endothelium is reduced in older people, an effect which contributes to decreased SkBF (Holowatz et al., 2006). While folic acid supplementation may potentially rescue some of the age-related declines in vasoreactivity (Stanhewicz et al., 2017), 6-weeks supplementation had no impact on SkBF or *T*_*c*_ during whole body heat stress in older adults (Gagnon et al., 2018). The above data pertains to elderly individuals, and as such cardiovascular perturbations are likely to be present but less severe in healthy workers <60 years of age.

### Matched Groups

An early study compared heat stress responses in young (19−31) and older (39−45) miners who performed stepping exercise over 4 h (Hellon et al., 1956b). The authors found similar *T*_*c*_ responses the first 3-h, after which the *T*_*c*_ was only 0.3°C higher in the older group. There were greater levels of cardiovascular strain in the older adults, marked by a 10 b⋅min^–1^ higher HR during work from 40 min to the end of the trial. Consequently, the older adults worked closer to their age-predicted maximum HR by ∼14%.

In the last decade, numerous studies have compared heat stress responses in well-matched participants of different age groups (Anderson and Kenney, 1987; Kenney, 1988; Kenney and Anderson, 1988; Tankersley et al., 1991; Larose et al., 2013, 2014; Stapleton et al., 2015; Kenny et al., 2017). In dry heat, older people store more heat due to reductions in evaporative heat loss, but the difference is proportional to the metabolic heat load. At heat loads > 325 W, sweat evaporation was reduced by ∼14% in those aged 58 compared with well-matched young adults in a hot dry environment (Stapleton et al., 2015). This resulted in greater levels of whole-body heat storage, and an increased *T*_*c*_. During cycling at 400 W, age-related decrements in sweat loss occurred as early as age 40 compared with young adults (Larose et al., 2013). In summary, there is an independent negative impact of age on sweat evaporation and heat storage during exercise in fully compensable environments.

High humidity decreases the proportion of sweat that evaporates into the environment to provide a cooling effect (Candas et al., 1979). Intuitively, there is less likely to be a different thermoregulatory response to humid heat between age groups, because young people cannot take advantage of their higher sweat rates. Indeed, at low to moderate intensity exercise, the difference in *T*_*c*_ between young and older groups is minimal in high humidity (Havenith et al., 1995b; Kenney, 1988; Kenney and Anderson, 1988; Tankersley et al., 1991; Larose et al., 2014), peaking at around ∼0.4°C after 1.5−2 h work (Kenney, 1988). However, studies do find blunted cardiovascular effector responses in older people during humid heat, such as reduced SkBF and cardiac output (Kenney, 1988; Tankersley et al., 1991; Havenith et al., 1995b). The maintenance of SkBF is particularly relevant for dry heat transfer, especially if ambient temperature < skin temperature.

### Population Averages

Compared with study designs that match young and old for all relevant characteristics, using unmatched participants better reflects the differences between age groups on a population level.

#### Fixed Work Rate

Havenith et al. (1995b) documented the relative importance of age compared with *V̇*O_2__*max*_, anthropometry, and adiposity on thermoregulatory and cardiovascular responses during cycling in humid heat, at equal absolute work rate. They showed that *V̇*O_2__*max*_, body mass and body surface area predicted the *T*_*c*_ and whole body sweat losses during exercise, but age did not. However, age *was* a strong predictor of the cardiovascular responses to humid heat, particularly SkBF which was lower with age, despite similar levels of cardiovascular strain (%HR_*max*_). While fitness (*V̇*O_2__*max*_ in L/min) was the primary indicator of HR, aging further reduced working HR in a non-linear fashion (Havenith et al., 1995b), likely due reduced beta-adrenergic responsiveness, calcium handling, and myocyte counts (Olivetti et al., 1991). The aging mediated reduction in HR and SkBF is due to structural and functional changes in the heart and cutaneous vasculature. Notable cardiac changes include reduced beta-adrenergic responsiveness, calcium handling, and myocyte counts (Olivetti et al., 1991). Age-related changes in vasodilatory function were described above. In the study of Havenith et al. (1995b), older people were working at a similar percentage of maximum HR compared with young adults of the same fitness level. Regardless of age, the fitness level was the main determinant of cardiac strain, in terms of both absolute HR and %HR maximum.

#### Relative Work Rate

Several studies have used an exercise intensity relative to fitness (i.e., %*V*O_2__*max*_) to compare thermometric and cardiovascular responses between unmatched young and older adults. Due to reduced fitness level, metabolic rate is normally lower in the older groups, meaning they produce less metabolic heat. Despite this reduced heat production, the negative aging effect on thermoregulatory function can result in similar *rates* of *T*_*c*_ rise in young and older people, despite the lower heat production in older individuals (Tankersley et al., 1991; Inbar et al., 2004). Consequently, working at a relative intensity yields a similar percentage of maximum HR across age groups, indicating that self-pacing can result in equivalent thermoregulatory and cardiovascular loads between age groups (Tankersley et al., 1991). In terms of protection from hyperthermia, the effectiveness of self-pacing will depend on the heat severity of the climate. Self-pacing may be less effective in uncompensable (high humidity) conditions, since required sweat evaporation for heat balance will typically be high regardless of any reduction of metabolic heat load (Sagawa et al., 1988; Dufour and Candas, 2007; Kenny et al., 2017). In less extreme, compensable conditions, self-pacing seems to be a good measure to prevent hyperthermia in older workers (Kalkowsky and Kampmann, 2006).

### Heat Tolerance

Tolerance times to heat are closely related to fitness (Cheung and McLellan, 1998), which is of relevance in this section because (i) young participants are generally more fit than aged participants (Betik and Hepple, 2008), and (ii) exercise training will infer greater tolerance to heat in an older population (Ho et al., 1997). On a population level, older people appear to be less vulnerable to heat if they work at an intensity relative to their fitness. At a fixed exercise intensity, older people are more vulnerable to heat on a population level. Using ROC curve analysis, Flouris et al. (2018b) show that age is a predictor of the heat stress response to fixed pace exercise. For heavy work in males, they suggest those over the age of 52 years are more likely to have a higher *T*_*c*_ than those below this age. For females performing moderate intensity work, they suggest a threshold of 56 years.

### Summary

Age can have a strong impact on the heat stress response on a population level, but this is primarily linked to reduced cardiovascular fitness. In matched groups, age has a moderate impact on the heat stress response. While exercise training in older people helps maintain cardiovascular responses to heat, reductions in sweat output are apparent at moderate to high heat loads. In a compensable environment, older people are expected to show lower levels of sweat evaporation, and thus a higher level of heat gain at moderate to heavy workloads. In an uncompensable environment, younger people cannot take advantage of a greater delivery of heat to the skin surface for cooling and may store heat at the same rate as older people. In general, absolute *V̇*O_2__*max*_ peaks at ∼20−29 years and declines as a function of age thereafter. Because tolerance to heat is largely dependent on the aerobic fitness level, one can expect a reduced performance in the older workforce. If older people can self-pace, the risk does not appear to be significant. Comparative heat stress responses between young and older people are shown in Table 7.

**TABLE 7:** Overview of data relating to the effect of age on the heat stress response.

Source	n	Younger group age (y)	Older group age (y)	Fitness matched?	Condition(s)	Work-type	Sweat rate		Baseline Core temperature (°C)	Peak Core temperature (°C)	Cardiovascular strain
Hellon et al., 1956b	36	19−31	39−45	No	38°C/52%	Step exercise with work/rest cycles	↓ 30%		↓ 0.1°C	↑ 0.2°C	↑ by ∼10 b/min throughout the work/rest cycles
Lind et al., 1970	12	22−31	39−53	No	25°C/64%	8-h exposure comprising work/rest cycles. Activity simulated manual labor	↓ 4% (NS)		−	↑ 0.1°C	Absolute heart rate higher in young, but%HR_*max*_ similar
					36°C/64%		↓ 8% (NS)		−	↑ 0.23°C	
Drinkwater and Horvath, 1979	38	12−68 (heterogeneous sample of women). 20 vs. 60 year olds used in this table		No	28°C/45%		Walking at 30−35% *V̇*O_2max_	NS	−	NS	Final%HR_*max*_ ↑ by ∼5−10% in all 3 conditions
					35°C/65%			↓ 33%	−	↑ 0.3°C	
					48°C/10%			↓ 14%	−	NS	
Drinkwater et al., 1982	20	38 ± 2	57 ± 2	No	40°C/30%		Passive exposure	NS	NS	NS	NS
Anderson and Kenney, 1987	16	20−30	52−62	Yes	48°C/14%		Walking at 40% *V̇*O_2max_	↓ 22%	NS	↑ 0.4°C	NS
Kenney and Anderson, 1988	16	20−30	52−62	Yes	37°C/60%		Walking at 40% *V̇*O_2max_	NS	NS	↑ 0.4°C	NS
Tankersley et al., 1991	13	24−30	58−74	Yes	30°C/55%		Cycling at 65% *V̇*O_2max_	NS	NS	NS	NS
Havenith et al., 1995b	56	20−73 (heterogeneous sample)		Yes	35°C/80%		Cycling at heat production of 300 W	See paper for regression equations			
								Related to *V̇*O_2max,_ not age.	−	Related to *V̇*O_2max,_ not age.	Related to age and *V̇*O_2max._
Inbar et al., 2004	16	23 ± 0.8	71 ± 1	No	41°C/21%		Cycling at 50% *V̇*O_2peak_	−	↓ 0.5°C	NS	↓ 20%
Larose et al., 2013	85	20−31	50−55	Yes	35°C/20%		Cycling at heat production of 400 W	↓ 9%	↓ 0.08°C	NS	−
Stapleton et al., 2015	20	21 ± 1	48 ± 5	No	40°C/15%		Cycling at heat production 300, 400, and 500 W	NS	NS	↑ 0.7°C	↑ 22% (only at 500 W)
			49 ± 5	Yes				NS	NS	NS	NS
Kenny et al., 2017	60	19−28	55−73	No	44°C, 30%		Passive exposure	↓ 27% (only at thigh)	−	↑ 0.2°C	%HR_*max*_ ↑ by ∼10%

*HR, heart rate; b/min, heart beats per minute; n, number of subjects; V̇O_2max_, maximal oxygen consumption; W/m^–2^, Watts per meters squared; y, years; NS, not statistically significant; T_c_, core body temperature; ↑, increased; ↓ decreased; −, not reported.*

### Practical Advice

• For fixed paced physical work in the heat, older people are more vulnerable to hyperthermia and reduced physical work capacity.
• If self-pacing is allowed, there should be no greater risk in older people compared with young people, if the workload is not heavy (see Table 1), and if the environmental heat is not extreme.
• Those over the age of 50 should be monitored closely upon initial exposure to heat stress. Those under this age are typically at less risk of heat injury.

## Sex

There are several factors relevant to thermoregulation that may differ between males and females. Studies on large subject numbers show that compared with males, females on average have a lower body mass and lower cardiorespiratory fitness (Kaminsky et al., 2015). In prior sections, these factors were shown to have a strong influence on heat stress vulnerability. Sex differences in thermoregulation have been reviewed previously (Burse, 1979; Havenith, 1985; Kenney, 1985; Kaciuba-Uscilko and Grucza, 2001), but there have been significant advances in this subfield in the last two decades, which are well summarized in a more recent review (Gagnon and Kenny, 2012a). The general conclusions from prior work are that males and females are mostly equal in thermoregulatory control if fitness and body composition are equal, despite small differences in sweat rates. This section will provide an update on the current state of knowledge regarding sex differences in heat stress responses.

### Population Averages

Early studies document the comparative responses of unmatched men and women to various types of heat exposures, representing population averages. The earliest comparisons were made in the 1940’s, documenting differential heat stress responses between men and women at rest (Hardy and Du Bois, 1940). Further comparisons demonstrate a delayed sweating onset and a reduction in maximum sweat rates in females (Bittel and Henane, 1975). During physical work, the impact of sex on the heat stress response seems to depend on the environment, fitness, and status of heat adaptation. During physical work in the heat, women initially suffered from greater *T*_*c*_’s and HR, but the differences subsided following a period of acclimation (Wyndham et al., 1965). In that study, the lighter mass for the women likely contributed to the faster rate of heat gain initially, while their greater sweating with heat adaptation later compensated (Havenith, 2001a). Using multiple regression, Havenith showed that gender was a predictor of the *T*_*c*_ response in dry and humid heat, but lost its predictive power when *V̇*O_2__*max*_ and body characteristics were added into the model (Havenith and van Middendorp, 1990). Therefore, the effect of sex as an independent variable is minimal in comparison to fitness and body characteristics. In unmatched participants, a heavy work rate caused a faster increase in *T*_*c*_ and HR in females compared with males (Gagnon et al., 2009). In that study, the increased heat vulnerability of the females is explained primarily by their lower body mass, but their lower fitness may have also contributed. On a population level, sex impacts heat vulnerability, owing primarily to the differences in fitness and body characteristics (Havenith and van Middendorp, 1990; Gagnon et al., 2009).

Evidence shows a reduced sweat output in females compared with males. Women have been shown to exert sweat rates as low as 30% to that of males, with the differences increasing as a function of the heat stress severity (Hardy and Du Bois, 1940; Wyndham et al., 1965; Hertig, 1971; Gagnon and Kenny, 2012b; Notley et al., 2017). Importantly, large differences in sweat output can also be due to women working at a lower rate of heat production during relative intensity work, due to their lower fitness level (Smith and Havenith, 2012). The importance of a lower sweat rate depends on the environmental humidity. In a dry environment, women typically show higher rates of *T*_*c*_ rise than men due to their reduced sweat evaporation (Shapiro et al., 1980; Frye and Kamon, 1981). However, greater sweat rates in males can cause a higher *T*_*c*_ and HR in uncompensable environments, due to faster rates of dehydration (Avellini et al., 1980; Shapiro et al., 1980; Havenith, 1985; Kenney, 1985). Importantly, the differences in sweat output between males and females are abolished after heat acclimation (Frye and Kamon, 1981).

### Matched Individuals

Compared with females of equal fitness, males showed a greater sweat rate and a lower *T*_*c*_ rise during treadmill walking in extreme dry heat at the same relative intensity (Frye and Kamon, 1981). The females also showed a greater HR in that study by 10−15 b⋅min^–1^, implying reduced physical work capacity during self-paced work. It is important to note that the sexes were only matched for fitness, not size, such that the males had a greater body mass and surface area, which are protective against rises in *T*_*c*_ and HR (Havenith and van Middendorp, 1990; Havenith et al., 1995a). However, the differences were abolished once both groups were acclimated. Cycling at a heat production of 500 W in dry heat, females matched for body characteristics and fitness had a lower sweat output for a given change in body temperature (Gagnon and Kenny, 2011). Here, the males activated heat loss responses (evaporation and cutaneous blood flow) at a lower body temperature compared with females, resulting in a lower end-exercise *T*_*c*_. The mechanism behind these responses are not fully elucidated, but recent evidence suggests that this may be due to differences in maximal sweat gland output (Gagnon et al., 2013a). Females seem to have a lower maximum sweat gland output compared with males, which means they compensate by activating a greater quantity of sweat glands. When the activated number of sweat glands reaches its maximum (i.e., a mean body temperature increase of ∼1°C), the higher maximal sweat gland output in males elevates sweat rate for the same mean body temperature (Gagnon and Kenny, 2012b; Gagnon et al., 2013a). An independent effect of sex on the heat stress response may only appear at heat loads > 250 W⋅m^–2^ (Gagnon and Kenny, 2012b).

### Menstrual Cycle

There are detectable differences in *T*_*c*_ throughout the menstrual cycle, specifically between the pre-and post-ovulation phases. Since the thermogenic hormone progesterone is released subsequent to ovulation, there is typically an increase in resting baseline *T*_*c*_ of ∼0.5°C (Kenshalo, 1966). Many studies were conducted in the late 1960’s to determine the impact of the menstruation on the heat stress response. Early work was equivocal, either finding a lower *T*_*c*_ “set-point” for the onset of sweating pre-ovulation (Haslag and Hertzman, 1965; Bittel and Henane, 1975; Stephenson and Kolka, 1985), or finding no meaningful differences in the *T*_*c*_ and sweat relation (Senay, 1973; Wells and Horvath, 1973). Based on a recent analysis, the weight of the evidence suggests that menstrual phase *does* alter the *T*_*c*_ onset thresholds for sweating and vasodilation, with delays up to 0.5°C in the luteal phase compared with the follicular phase (Charkoudian et al., 2014). Of note, mild rises in *T*_*c*_ have been shown in the luteal phase with combined use of oral contraceptives (Lei et al., 2019). However, measurement of onset thresholds need to be conducted under well-controlled conditions, since their effects are quite small and can be negated by other factors, such as time of day (Stephenson and Kolka, 1985). Also, onset thresholds for vasodilation and sweating are typically conducted in one limb and might well be compensated for in other body areas. Therefore, it is important to consider whole body heat stress responses to physical activity in the heat to determine the true relevance of the menstrual cycle. When the female response to 2-h extreme dry heat was compared at the three menstrual phases, there were no significant differences in *T*_*c*_, skin temperature, or body heat content (Wells and Horvath, 1973). It was also shown that differences between pre-and post-ovulation are not affected by heat acclimation. During exercise at a fixed rate of heat production, there were no differences in *T*_*c*_ or *T*_*sk*_ when women exercised at the follicular or luteal phase of menstruation. Moreover, there were no differences in biophysical parameters such as *E*_*max*_, required evaporation, and whole-body heat storage (Notley et al., 2019a). In summary, menstrual phase has a marginal impact on the heat stress response and is unlikely to dictate independently whether an individual is vulnerable to heat stress.

### Summary

Sex has a moderate impact on the heat stress response which becomes minor if body characteristics and fitness factors are accounted for. The impact of sex on hyperthermia and work capacity are mostly relevant during heavy work in compensable environments. Compared with men of equal fitness and body composition, women may have a higher HR and reduced capacity for sweat evaporation at heavy workloads, but the differences disappear if both groups are heat acclimated. If the heat load is strong enough, this can result in a greater rate of heat storage and thus an elevated *T*_*c*_ in unacclimated females. Menstruation appears to affect resting *T*_*c*_ but does not influence the rate of heat storage or the threshold for sweating onset. The effect of sex on thermoregulatory responses to heat are shown in Table 8.

**TABLE 8:** Overview of data relating to the heat stress responses in women when compared with men.

Source	n	Fitness matched↑	Condition	Work-type	Sweat threshold (°C)	Sweat rate	Baseline Core temperature (°C)	Peak Core temperature (°C)	Cardiovascular strain
Wyndham et al., 1965	56	No	34°C/90%	Step test at 1 l/min *V̇*O_2_		↓ 30%	NS	↑ 0.4°C	↑ 20 b/min
Bittel and Henane, 1975	14	No	45°C/30%	Resting	∼5 min onset delay	−	NS	↑ 0.4°C	−
						See paper below for regression equations			
Havenith and van Middendorp, 1990	26	Yes	34°C/80%	Relative intensity cycling	−	There was an effect of gender on the heat stress response, but this was due to differences in body characteristics.			
			45°C/20%						
Frye and Kamon, 1981	8	Yes	48°C/14%	Relative intensity cycling. 3 h at 30% *V̇*O_2max_	NS	↓ 34%	NS	↑ 0.44°C	↑ 12 b/min
Gagnon et al., 2009	12	No	30°C/30%	Cycling exercise at 500 W	−	−	−	↑ 0.50°C	↑ but no data provided.
Gagnon and Kenny, 2011	18	No	35°C/12%	Relative intensity cycling for 90 min. 50% *V̇*O_2max_	NS	↓ 21%	NS	NS	−
				Fixed intensity cycling for 90 min. Heat production equal to 500 W	NS	↓ 33%	NS	↑ 0.46°C	−

*b/min, heart beats per minute; n, number of subjects; V̇O_2max_, maximal oxygen consumption; NS, not statistically significant; ↑, increased; ↓ decreased; −, not reported.*

### Practical Advice

• On a population scale, males are more suited to hot work compared with females. However, the minimum criteria for hot work should be initially based on fitness and age, not sex.
• Females will typically be more at risk of hyperthermia if the heat load is high (see Table 1).
• Males are likely to dehydrate faster in uncompensable heat stress, due to higher sweat output.
• Once fully acclimatized to the heat, the heat stress response between matched males and females is similar.

## Chronic Health Conditions

Chronic health conditions have an important impact on the human heat stress response. Addressing all health conditions is beyond the scope of this review because our findings are applied to those who perform physical work in the heat. We primarily focus on diabetics due to its current and future global prevalence (Roglic, 2016), and available research investigating its impact on whole body heat stress responses. We discuss hypertension and cardiovascular diseases more briefly because the proportion of the population with clinically relevant hypertension or heart disease performing physical work in the heat is likely to be small, and applies mostly to the elderly population (Kenney et al., 2014). Moreover, research investigating whole body physiological responses to heat in those with these conditions is sparse, and in some cases absent entirely. Nonetheless, understanding the role of cardiovascular disease in vulnerability to heat is relevant for scenarios in which people with these underlying conditions still perform work in the heat.

### Diabetes

The World Health Organization estimates that 422 million adults have diabetes, the majority of which have type 2 diabetes. The global prevalence of diabetes has nearly doubled since 1980, rising from 4.7 to 8.5% of the adult population (Roglic, 2016), and prevalence rates may reach an astonishing 33% by 2050 (Boyle et al., 2010). With diabetes being the most prevalent morbidity present in the population, it is pertinent to address whether thermoregulatory function is impaired in these individuals. We should note that more in-depth reviews are available specific to diabetes and thermoregulation, for the interested reader (Yardley et al., 2013; Kenny et al., 2016). Our aim in this section is to give a concise summary of the primary information of relevance for employers and policymakers.

#### Local SkBF

Thermal physiologists have taken interest in diabetics because local SkBF and sweating are negatively correlated with the level of glucose control (Wigington et al., 2004; Petrofsky et al., 2009; Brugler et al., 2011). At a normal *T*_*c*_ of ∼37°C, there is very little difference in SkBF between diabetic and non-diabetic participants, but diabetics have shown up to a 50% reduction in local SkBF stimulated by heat or vasodilatory compounds (Rendell et al., 1989; Brugler et al., 2011; Fujii et al., 2018). The link between diabetes and a reduced SkBF is coined diabetic cutaneous microangiopathy and can affect both type 1 (T1DM) and type 2 (T2DM) diabetes mellitus sufferers. In T1DM, there is no release of C-peptide which is produced in pancreatic β cells, and the peptide has known roles in maintaining microvascular blood flow (Forst et al., 1998; Forst and Kunt, 2004). In T2DM, the reductions in SkBF may be due to a reduced nitric oxide bioavailability (Williams et al., 1996; Beer et al., 2008), which is worsened by the presence of atherosclerotic plaques (Watson et al., 2003; Kawashima and Yokoyama, 2004). There may be additional factors at play, and the interested reader is directed to two reviews for further reading (Ngo et al., 2005; Kenny et al., 2016).

#### Whole Body Responses

The above evidence is based on local SkBF measurements with laser Doppler, but until recently it was unknown whether they translate into meaningful whole-body responses. The evidence thus far is equivocal and seems to depend on the severity of the condition and physical fitness of the individual. For instance, young-adult recreationally active T1DM sufferers were compared against well-matched healthy controls during 1-h cycling exercise at 400 W metabolic heat production, and the thermoregulatory responses were similar between both groups (Stapleton et al., 2013). When the heat load was increased to 500 W, T1DM sufferers exhibited lower sweat rates in the forearm and chest, which in turn led to an increased *T*_*c*_ by up to 0.5°C (Carter et al., 2014). The findings were repeated in a later study which showed impaired thermoregulation during exercise in T1DM patients (McGinn et al., 2015). Most recently, a study demonstrated reduced evaporative heat loss and higher *T*_*c*_ in young adults with T1DM but only during heavy work (Notley et al., 2019c). For T2DM sufferers working at a high metabolic rate for 1-h in mild heat, an increased heat storage rate was documented due to a lower rate of evaporative heat exchange, compared with health matched controls (Kenny et al., 2013). Overall, it seems that recreationally active diabetes sufferers can show impairments in heat loss if the workload is heavy. Whether or not prolonged work at a lower rate of heat production is dangerous for diabetics has not been investigated.

### Cardiovascular Disease

Cardiovascular disease is a broad term that can encompass several conditions such as chronic heart failure, coronary and valvular heart disease, cardiomyopathy, congenital heart defects, and cerebrovascular and peripheral vascular disease (Kenny et al., 2010). The majority of deaths during heat waves are attributed to cardiovascular issues, and are predominant in the elderly population (Conti et al., 2005; Kenney et al., 2014). During heat stress, the elevated risk of death in those with cardiovascular disease has been described previously (Kenney et al., 2014). Although risk of death is higher in those with cardiovascular disease, its impact on hyperthermia risk and HR during work in the heat is less clear due to the paucity of evidence. Patients with congestive heart failure had similar *T*_*c*_ and HR responses to passive heat stress compared with healthy controls, despite lower SkBF (Cui et al., 2005). In that study, those in the congestive heart failure group did not discontinue any medication, which we consider a strong reflection of real-world responses. In a later study, it was shown that patients with chronic heart failure showed no difference in *T*_*c*_ and HR, with no reduction in sweat rate, again despite a lower SkBF (Cui et al., 2013). That work used a water perfused suit model which limits most excreted sweat from evaporating, making comparisons in *T*_*c*_ problematic. During 3-h exposure to mild heat stress in a climatic chamber, those with ischemic heart disease experienced similar thermoregulatory responses to healthy controls (Andersen et al., 1976). During cycling exercise in mild heat, heart failure resulted in a faster *T*_*c*_ rise compared with control participants, either at relative (Balmain et al., 2016) or fixed intensity work (Balmain et al., 2018a). The faster increase in *T*_*c*_ was primarily due to a reduced SkBF downstream of attenuated cardiac function, and folic acid supplementation (which enhances nitric oxide bioavailability) was shown not to enhance this response in a later study, despite increase vascular function in general (Balmain et al., 2018b). Finally, the thermoregulatory responses to physical activity in the heat was not affected by previous coronary artery bypass surgery, compared with healthy controls (Walsh et al., 2002). Overall, cardiovascular disease is not a strong *independent* predictor of the heat stress response in the working population. However, individuals with cardiovascular disease are likely to present with very low fitness levels, which is a major risk factor for hot work.

### Hypertension (High Blood Pressure)

Hypertension is a long-term medical condition characterized by a permanent elevation of peripheral resistance. Hypertension decreases resting SkBF and produces a shallower slope between SkbF and increased *T*_*c*_ (Kenney et al., 1984; Carberry et al., 1992). As noted in the clarification of terms section, such changes in SkBF do not necessarily translate into greater heat vulnerability during physical work. When well matched normotensive and hypertensive participants are compared during physical work in the heat, their *T*_*c*_ and HR responses are similar (Kenney and Kamon, 1984; Fonseca et al., 2015). As noted by Kenny et al. (2010), the use of antihypertensive medications (diuretics, vasodilators, β-blockers) may independently increase susceptibility to heat stress issues. Similarly to cardiovascular disease, hypertension has a marginal *independent* effect on the heat stress response. However, individuals with hypertension are likely to present with lower fitness levels, which is a major risk factor for hot work.

#### Summary

Diabetes can have a moderate impact on heat stress responses, depending on the heat load and the disease severity. There is strong evidence that SkBF can be reduced in T1DM and T2DM induced by local heating or vasodilator compounds. During whole-body heat stress, impairments in heat loss have been shown in unfit T2DM sufferers and recreationally active T1DM patients if the level of heat production is high (i.e., 500 W). The independent effect of cardiovascular disease and hypertension is marginal during whole body heat stress, but secondary risk factors (i.e., very low fitness levels) are likely to be present in these populations.

#### Practical Advice

• Based on the literature available, people with diabetes may be at risk during hot work.
• People with diabetes who have good glucose control should not be considered more at risk if they meet fitness standards and can acclimatize to the work.
• Cardiovascular disease and hypertension do not increase vulnerability to heat *per se*, but secondary risk factors such as very low fitness level increase susceptibility to heat stress.

## The Relative Influence of Individual Characteristics

The relative influence of each physical characteristic on the *T*_*c*_ response and physical work capacity during heat stress is displayed in Figure 1. The *T*_*c*_ figure is most relevant from a health and safety perspective, as the International Standard states that average *T*_*c*_ should not exceed 38°C during a typical working day for a group of workers. The key factors for *T*_*c*_ control during heat stress are acclimation status, body mass, and fitness. The work capacity figures focus on how each factor influences work output in the heat, based on the assumption that work output is regulated by the HR, as has indeed been shown in the field (Mairiaux and Malchaire, 1985; Brake and Bates, 2002; Miller and Bates, 2007; Miller et al., 2011).

## Conclusion

The present review has determined which individual characteristics are most relevant during physical work in the heat, in the context of health and work capacity. It should be noted that several other factors can independently influence thermophysiological responses to heat, such as dehydration (Cheuvront and Kenefick, 2014), some medications (Martin-Latry et al., 2007), and psychological stress (Boulant, 2010). The present review is written with the assumption that workers are hydrated, not on prescription medication, and are free of severe psychological distress. There is an urgent need to explore the interaction between multiple factors, and the time dependency for certain factors to take effect.

## Author Contributions

JF drafted versions of the manuscript with input and revisions from GH, SH, and AL. All authors contributed to the article and approved the submitted version.

## Conflict of Interest

The authors declare that the research was conducted in the absence of any commercial or financial relationships that could be construed as a potential conflict of interest.

## Abbreviations

*A*_*D*_:mass: skin surface area to mass ratio
BMI: body mass index
*E*_*max*_: maximum evaporative potential
*E*_*req*_: required evaporation for heat balance
HSP72: heat shock protein 72
ISO: International Standardization Organization
kPa: water vapor pressure
NBC: clothing, nuclear, biological, and chemical protective clothing
ROC: receiver operator characteristic
SkBF: skin blood flow
T1DM and T2DM: type 1 or 2 diabetes mellitus
*T*_*c*_: core temperature
*V̇*O_2max_: maximal oxygen consumption
W: watts
WBGT: wet-bulb globe temperature
WHO: World Health Organization.
